# Theoretical Analysis and Numerical Simulation Research on the Response of Inertial Switches

**DOI:** 10.3390/mi16040459

**Published:** 2025-04-13

**Authors:** Shaokang Cui, Jiachen Hao, Sen Wang, Fukai Wu, Pengzhao Xu

**Affiliations:** 1 Xi’an Institute of Electromechanical Information Technology, Xi’an 710065, China; 2 School of Mechanical Engineering, Northwestern Polytechnical University, Xi’an 710072, China; 3 Science and Technology on Electromechanical Dynamic Control Laboratory, Xi’an 710065, China

**Keywords:** theoretical analysis, numerical simulation, inertial switches, dynamic response

## Abstract

This paper focuses on inertial switches, which are vital in multiple fields. A theoretical analysis model based on the spring-mass model was proposed, aiming to acquire the mechanical performance of the inertial switch under different loading conditions and reveal the influence of design parameters on its dynamic response. Moreover, a numerical simulation model was established using ADAMS 2019 software. Through a series of orthogonal simulations, the effects of the overload peak value and pulse width on the switch’s characteristics were studied. The comparison between theoretical predictions and numerical results shows good agreement. Furthermore, based on numerical results, binary phase diagrams of the peak value and pulse width were obtained, determining the critical curves involving the overload peak value and pulse width, which provides guidance for the design of inertial switches.

## 1. Introduction

Inertial switches play a crucial role in numerous fields, such as aerospace [1,2,3], military [4,5,6,7,8,9], automotive safety [1,10,11,12], and industrial automation [13,14,15]. With the development of science and technology, the requirements for their response performance are becoming increasingly stringent. It is of great importance to accurately understand and optimize the response characteristics of inertial switches. In the field of military research, the inertial switch is an important component commonly used in penetration fuzes, mainly used to control the working state of the first-stage electro-explosive device circuit in the fuze explosion sequence. It closes by using the reaction force or forward-rushing inertial force when the carrier impacts the target, turning on the fuze firing control circuit to input energy into the first-stage electro-explosive device and triggering the initiation element. It is an important component for the fuze to achieve the functions of contact detonation or self-destruction upon landing. This has extensive requirements and applications in weapon ammunitions such as radio fuzes and electromechanical fuzes. At present, although the size parameters of the inertial switches used in various fuzes are different, they generally adopt the structural form of the PF-1 radio fuze for projectiles. The critical overload values for these switch closings varies from 100 g to several hundred g with a working time of hundreds of microseconds. The switch consists of a metal shell, a safety spring, an impact rod, an insulating sleeve, and a conductive sleeve. The metal shell, safety spring, and impact rod form one pole of the percussion switch, and the conductive sleeve forms the other pole. The two poles are separated under normal circumstances. The working principle of the switch is that the impact rod is pressed tightly in the safe position by the spring, and when the target is impacted, the switch closes under the reaction of inertial force.

Many studies have been carried out to investigate the response of the inertial switch. Fang et al. [4] proposed an axially installed inertial trigger switch with a weight-adding column based on the PF-1 inertial switch. In this switch, a weight column was added below the original inertial impact rod and the dynamic response of the switch was then tested through an experimental method. The results showed that the introduction of the weight column increased the critical overload peak value of the switch. Ning et al. [5] designed a cantilever beam-type unidirectional high-overload inertial switch for fuzes based on the PF-1 inertial switch and verified the insensitivity and instantaneity of the switch through experimental methods. Calculations and experiments showed that when the switch was placed at the front end of the fuze, it would survive the ballistic environment with insensitivity requirements. At the end of the ballistic trajectory, when the fuze impacted a hard target, the switch could close quickly. Furthermore, Frobenius et al. [6] from the Westinghouse Research Laboratory (WRL) in the United States proposed an all-metal cantilever beam-type MEMS inertial switch for the first time. It consisted of a flexible metal cantilever beam and a corresponding contact plate. The metal cantilever beam was used as an elastic structure and a sensitive mass. When subjected to an external overload, the cantilever beam experienced the overload and deformed. When the overload exceeded the critical value, the cantilever beam was in contact with the contact plate and output a switch-closing electrical signal. Moreover, Noetzel and Tönnesen [7,8] in Germany developed a horizontally driven MEMS inertial switch composed of a mass block–cantilever beam system, and they found that by moving the position of the mass block from the end of the cantilever beam to the middle of the cantilever beam, that is, using the softer end of the cantilever beam instead of the rigid mass block to contact the fixed electrode, the bounce-back effect could be reduced and the contact time would then be extended.

On the other hand, in terms of theoretical analysis, the switch had commonly been simplified as a single-degree-of-freedom spring-mass system and the dynamic equation under the action of inertial force had been established [6,7,8]. In this model, the mass block moving electrode is simplified into a mass-spring-damper system that is composed of a mass block with mass *m* and a massless spring with a spring constant *k* and damping coefficient *c*. When the external overload *a* is large enough so that the relative displacement of the mass block *z* reaches or exceeds the gap *d* between the two electrodes, the switch closes and provides a conduction signal to the external circuit. However, rigid electrodes are widely employed in this model, leading into a rigid contact. Therefore, under the reaction of overload, the mass block rebounds immediately after a violent collision with the electrode, resulting in a very short contact time (less than 10 μs), which directly affects subsequent signal processing and the reliability of the device. Consequently, in order to improve the contact effect and extend the switch contact time, many scholars have proposed solutions, such as using movable contacts, carbon-nanotube-contact electrodes, and flexible electrodes to extend the switch contact time. Furthermore, authors conducted comprehensive research on the theory of inerters and inerter-based vibration isolation in this work [16]. They delved into the fundamental principles of inerters, which are mechanical two-terminal devices that produce a force proportional to the relative acceleration between their terminals. The theoretical aspects might cover the mathematical models used to describe inerter behavior, such as the equations that relate force, acceleration, and inertance. Additionally, the review might touch on the theoretical development of inerter-based control strategies and how they contribute to optimizing the performance of vibration isolation systems. Overall, this paper provides a thorough theoretical foundation for understanding inerters and their role in vibration isolation, which can guide the design and application of inerter-based devices and systems.

Furthermore, the literature includes multi-faceted research on the challenges and breakthroughs of inertial switches in practical applications. In terms of challenges, inertial switches face issues such as environmental interference, reliability, size limitations, power consumption, compatibility with other devices, and meeting the requirements of special application scenarios, such as high-g tolerance, shock resistance, and electromagnetic interference immunity in military applications [17,18]. In terms of breakthroughs, a number of research achievements have been made. For example, in 2019, a study used SiC whisker and SU-8 composite-reinforced materials to increase the Young’s modulus of the MEMS inertial switch by 12% and the threshold acceleration by 20% [19]. In 2024, a team proposed a tunable multi-threshold inertial switch based on a cantilever-type micro-beam, which can classify acceleration and save energy in the untriggered state [1]. In the same year, there was also a micro-electromechanical structure inspired by origami. Combined with the skin, it can achieve a bistable self-sensing deformation mode, which can transmit stimuli in the form of normal or shear forces and can be integrated with inertial sensors of smartphones [20]. In 2025, a self-powered sensor based on an asymmetric bistable oscillator was proposed, realizing the dual-mode functions of energy harvesting and impact sensing, and having strong anti-false-triggering performance [21]. Regarding structural design for special applications, in high-inertial-impact environments like those in ammunition systems, research on MEMS inertial detection and drive-integrated mechanisms is ongoing. This aims to explore real-time recognition principles of double-thresholds for high-impact acceleration amplitude and pulse width and analyze microscopic collision mechanisms under high-dynamic conditions [22]. In addition, there are research reviews on aspects such as the sensitive direction, threshold acceleration, contact effect, and threshold accuracy of inertial switches, as well as discussions on how to improve the performance of inertial switches in different application fields such as automotive electronics, the Internet of Things, and military, providing theoretical support and technical guidance for the further development and widespread application of inertial switches [23,24,25].

In the current research landscape of inertial switches, a comprehensive review reveals a significant lacuna. While numerous experimental and some simulation-based studies have been conducted, there is a conspicuous lack of in-depth theoretical analysis. Existing works mainly focus on the performance testing of inertial switches under various conditions or the optimization of their structural parameters through numerical simulations. However, without a solid theoretical foundation, it is arduous to precisely predict the behavior of inertial switches in different engineering scenarios. Against this backdrop, our study aims to fill this crucial gap. By conducting a detailed theoretical analysis of inertial switches, we seek to provide a robust framework that can guide engineering design. Our approach not only combines theoretical modeling with experimental validation but also incorporates innovative concepts to enhance the performance of inertial switches. This research is expected to not only contribute to the academic understanding of inertial switches but also have far-reaching implications for their practical applications in various fields.

In this work, the dynamic response of inertial switches was studied using theoretical and numerical methods. First of all, the theoretical model was established based on the spring-mass system, and the displacement of the mass was then obtained. Moreover, the effects of the peak value and pulse width of overload on the dynamic performances of the switch were analyzed in detail, combined with a numerical simulation. As a consequence, the displacement of the mass was calculated and compared to the theoretical predictions, and it was found that the numerical and theoretical results agree with each other. Finally, some important conclusions are summarized based on the results and discussion.

## 2. Theoretical Modeling

**Figure 1:**
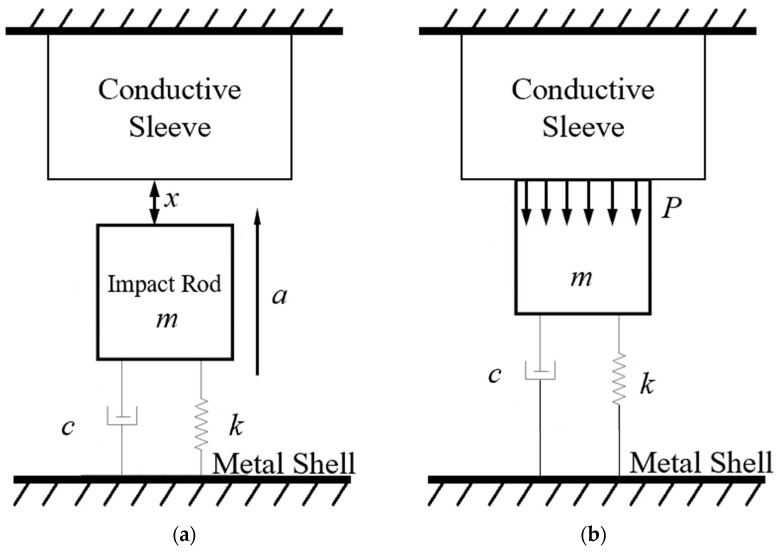
Physical model of the inertial switch: (**a**) before impact, (**b**) during impact.

The physical model of the inertial switch is a typical spring-mass mechanical system, as shown in Figure 1a. Under the axial overload *a*, the impact rod with mass *m* impacts the conductive substrate, as shown in Figure 1b. The resulting contact force and displacement are represented by *P* and *u*, respectively. It is assumed that the impact follows the rigid-body kinematics theory, except for the spring. Therefore, the dynamic equation of the switch can be expressed as:

(1) $$m\ddot{u}+c\dot{u}+ku=ma$$

where *c* is the damping coefficient of the system; *k* is the stiffness coefficient of the spring. It can be seen from Equation (1) that when the external overload *a* is large enough so that the relative displacement *x* of the mass block reaches or exceeds the gap *d* between the two electrodes, that the switch would close and provide a conduction signal to the external circuit.

The critical overload is the minimum value required to turn on the inertial switch, meaning the displacement of the mass block reaches the gap *d* between the two electrodes, denoted as *a_c_*. During the filed application process, the experienced excitation of the switch in the environment could generally be approximated as a half-sine overload, which is the most common form of impact overload. Therefore, unless otherwise specified, this is the scenario mainly discussed in this paper. Specifically, the applied overload is a half-sine curve with a peak value of *a*_0_ and pulse width of *t*_0_, and its mathematical expression is:

(2) $$a\left(t\right)=\left\{\begin{array}{c} a_{0}\sin\omega_{0}t\;\;\;\;\;\;t<t_{0}\\ \;\;\;\;\;\;\;\;\;\;0\;\;\;\;\;\;\;\;\;\;\;\;\;t>t_{0} \end{array}\right.$$

where *t*_0_ = π/*ω*_0_, and *ω*_0_ is the angular frequency of the half-sine impact overload. At the same time, it is assumed the displacement and velocity of the system at the initial time *t* = 0 are *u*(0) = *u*_0_ and $\dot{u}\left(0\right)={\dot{u}}_{0}$, respectively.

To simplify the analysis process, the influence of damping on the system response is temporarily ignored. Substituting Equation (2) into Equation (1) and considering the initial conditions, we obtain:

(3) $$u=\left\{\begin{array}{c} u_{I}=u_{0}\cos\omega_{n}t+\frac{{\dot{u}}_{0}}{\omega_{n}}\sin\omega_{0}t+\frac{a_{0}}{\omega_{n}^{2}-\omega_{0}^{2}}\left(\sin\omega_{0}t-\frac{\omega_{0}}{\omega_{n}}\sin\omega_{n}t\right)\;\;\;t<t_{0}\\ u_{R}=u\left(t_{0}\right)\cos\omega_{n}\left(t-t_{0}\right)+\left(\frac{\dot{u}\left(t_{0}\right)}{\omega_{n}}-\frac{a_{0}\omega_{0}}{\omega_{n}\left(\omega_{n}^{2}-\omega_{0}^{2}\right)}\right)\sin\omega_{0}\left(t-t_{0}\right)t>t_{0} \end{array}\right.$$

where *ω*_n_ is the frequency of the mass-spring system, and *u_I_* and *u_R_* represent the initial and residual response of the system, respectively. According to Equation (3), the relationship between the dynamic response *u* of the system and time *t* can then be obtained, that is, the displacement–time history. The initial response, also called forced response, is the displacement–time history of the system during the loading time, and the residual response, also called free response, is the displacement–time history of the system after the loading disappears. If the frequency of the switch and the boundary conditions are given, the initial response and residual response of the switch can be calculated using Equation (3), then the maximum displacement of the switch can be obtained. When the maximum displacement is larger than the gap between the two poles, the switch closes; otherwise, it does not.

## 3. Numerical Model

**Figure 2:**
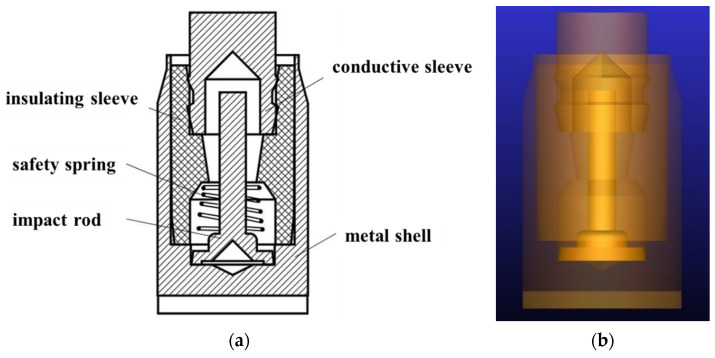
(**a**) PF-1 inertial switch, (**b**) kinematic model of the inertial switch.

The inertial switch mainly consists of a metal shell, an impact rod, an insulating sleeve, a conductive sleeve, and a safety spring, as shown in Figure 2a. The 3D model of the inertial switch was created in NX12.0 and then imported into the simulation software as shown in Figure 2b. The commercial software, ADAMS, was adopted to simulate the impact process to understand the dynamic characteristics of the inertial switch and evaluate important parameters such as the critical overload. In addition, the “Impact” contact algorithm was used between the impact rod and the conductive sleeve to improve the simulation accuracy, in which the contact force *P* was given by a function of these parameters:

(4) $$P=K\delta^{e}+C\dot{\delta }$$

where *K* is the contact stiffness coefficient, *δ* is the contact penetration depth, *e* is the stiffness exponent, and *C* is the contact damping coefficient. The contact *δ* can be obtained from empirical data and material tests. It is noted that the friction during the movement process is ignored compared with the inertial force. Initially, the gap between the impact rod and the conductive sleeve is 0.53 mm. That is, when the free-end radial displacement of the impact rod *D_p_* reaches the critical radial displacement of 0.53 mm, the impact rod contacts the conductive sleeve and the switch closes and conducts; otherwise, the switch does not close. The material of the impact rod is lead brass, with a density of 8.5 g/cm^3^ and mass of 0.067 g; the initial compression of the safety spring is 2.54 mm with a resistance force of approximately 0.147 N.

Furthermore, boundary conditions included the following:

(i) The fixed constraint was applied between the metal shell and the ground, between the insulating sleeve and the metal shell, and between the conductive sleeve and the insulating sleeve.
(ii) An overload curve that impacts along the radial direction was applied at the centroid of the impact rod.
(iii) A contact pair was applied between the impact rod and the conductive sleeve.

In terms of boundary conditions, these had been expounded in the article and were applied based on the actual physical structure model. In terms of mesh generation, the rigid-body dynamics analysis of ADAMS was employed, thus there is no need for mesh generation. Regarding the selection of the time step and contact stiffness, the end time was set to 2 ms with 200 steps, and the stiffness was set to the default value according to the research findings in the references [26,27,28].

## 4. Results and Discussion

### 4.1. Comparison Between Theoretical and Numerical Results

**Figure 3:**
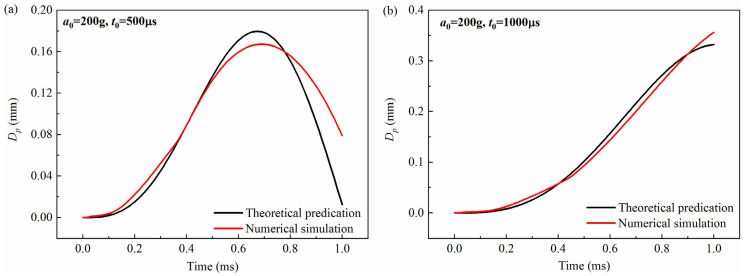
Comparison between theoretical prediction and simulation calculation results of the free-end displacement of the impact rod under different pulse widths when the peak value *a*_0_ = 200 g: (**a**) (*t*_0_ = 500 μs), (**b**) (*t*_0_ = 1000 μs). *D_p_* was the free-end radial displacement of the impact rod.

**Figure 4:**
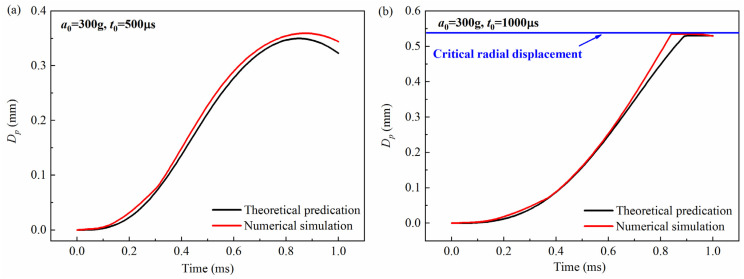
Comparison between theoretical prediction and simulation calculation results of the free-end displacement of the impact rod under different pulse widths when the peak value *a*_0_ = 300 g: (**a**) (*t*_0_ = 500 μs), (**b**) (*t*_0_ = 1000 μs). *D_p_* was the free-end radial displacement of the impact rod.

**Figure 5:**
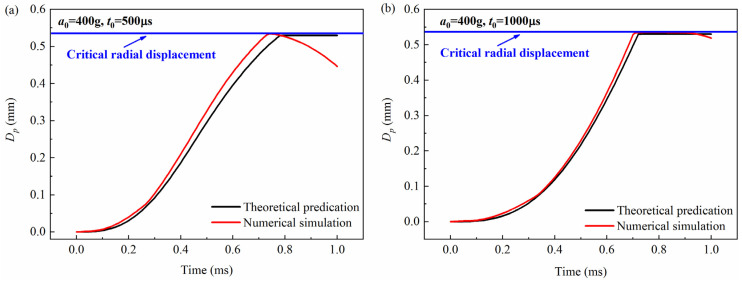
Comparison between theoretical prediction and simulation calculation results of the free-end displacement of the impact rod under different pulse widths when the peak value *a*_0_ = 400 g: (**a**) (*t*_0_ = 500 μs), (**b**) (*t*_0_ = 1000 μs). *D_p_* was the free-end radial displacement of the impact rod.

The theoretical prediction of the free-end displacement of the impact rod obtained from Equation (3) was compared with the numerical simulation, as shown in Figure 3, Figure 4 and Figure 5. It can be seen from the figures that for the peak values of 200 g, 300 g, and 400 g, and the pulse widths of 500 μs and 1000 μs, the theoretical calculations are in good agreement with the simulation results. To further verify the accuracy of the model, the maximum value of the free-end displacement of the impact rod *d_m_* and the corresponding response time *t_r_* were extracted and then compared with the results shown in Table 1 and Table 2. It can be seen from the tables that under different peak values (*a*_0_ = 200 g, *a*_0_ = 300 g, *a*_0_ = 400 g) and overload pulse widths (*t*_0_ = 500 μs, *t*_0_ = 1000 μs), the theoretical calculations and numerical simulations show good agreement.

### 4.2. Influence of Peak Value and Pulse Width of the Overload

**Figure 6:**
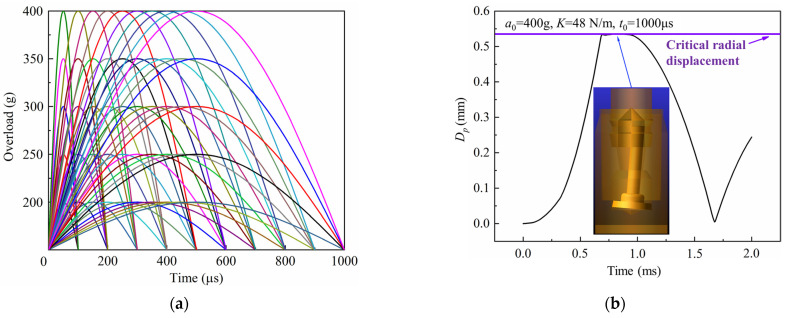
(**a**) Overload curves under some working conditions, (**b**) end-displacement curves of the inertial switch under typical working conditions.

In order to analyze the response of the inertial switch under different peak values and pulse widths of overload, an orthogonal simulation experiment was designed for calculation and analysis. Considering the working environment of the inertial switch, peak values of 200 g, 250 g, 300 g, 350 g, and 400 g, and pulse widths of 100 μs, 200 μs, 300 μs, 400 μs, 500 μs, 600 μs, 700 μs, 800 μs, 900 μs, and 1000 μs were selected, and all conditions are shown in Figure 6a. The total simulation time was 2 ms which was composed of a forced response of 1 ms and a free response of 1 ms. By extracting the calculation results, the history curves of the free-end displacement of the impact rod of the inertial switch versus time under typical working conditions were obtained, as shown in Figure 6b. In this figure, *a*_0_ is the peak value of the applied excitation, *K* is the spring stiffness coefficient, and *t*_0_ is the overload pulse width of the applied excitation. It can be seen from the figure that the inertial switch closes normally under this working condition.

**Figure 7:**
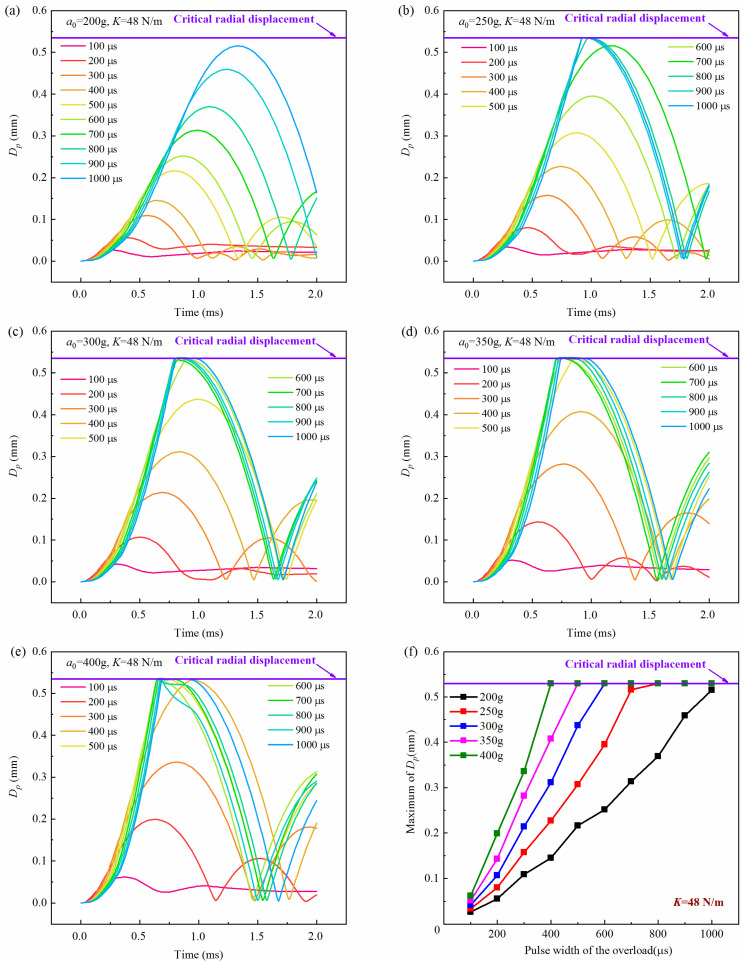
History curves of the free-end displacement of the impact rod of the inertial switch (*K* = 48 N/m) with time under different overload peak values with overload pulse width varying from 100 μs to 1000 μs: (**a**) *a*_0_ = 200 g, (**b**) *a*_0_ = 250 g, (**c**) *a*_0_ = 300 g, (**d**) *a*_0_ = 350 g, (**e**) *a*_0_ = 400 g and (**f**) relationship between maximum value of *D_p_* and overload pulse width. *D_p_* was the free-end radial displacement of the impact rod.

**Figure 8:**
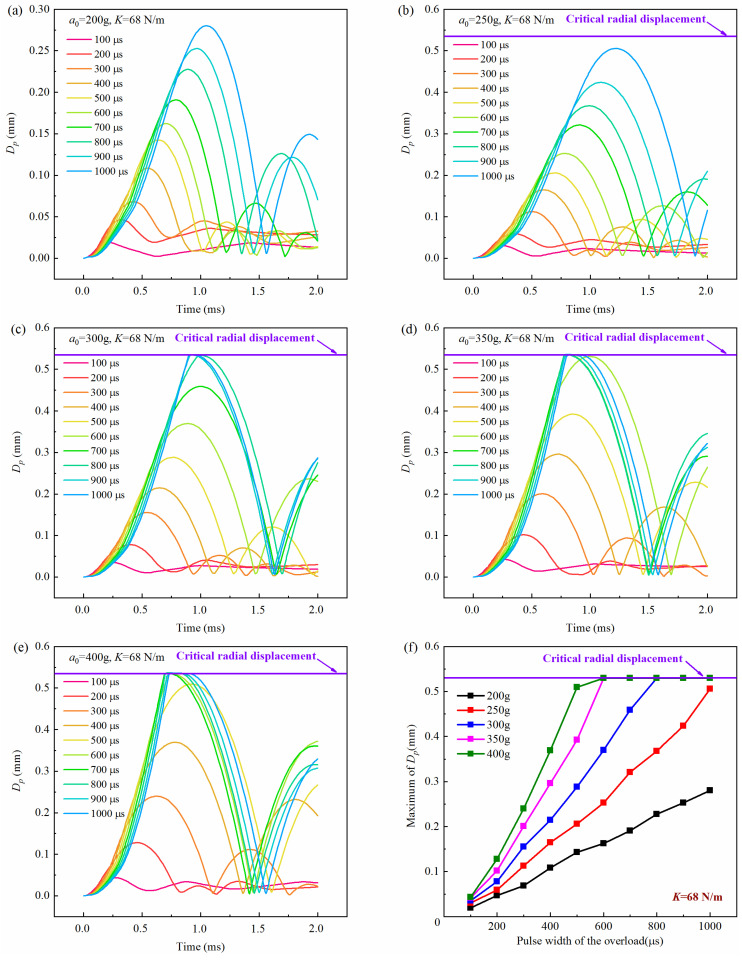
History curves of the free-end displacement of the impact rod of the inertial switch (*K* = 68 N/m) with time under different overload peak values with overload pulse width varying from 100 μs to 1000 μs: (**a**) *a*_0_ = 200 g, (**b**) *a*_0_ = 250 g, (**c**) *a*_0_ = 300 g, (**d**) *a*_0_ = 350 g, (**e**) *a*_0_ = 400 g and (**f**) relationship between maximum value of *D_p_* and overload pulse width. *D_p_* was the free-end radial displacement of the impact rod.

**Figure 9:**
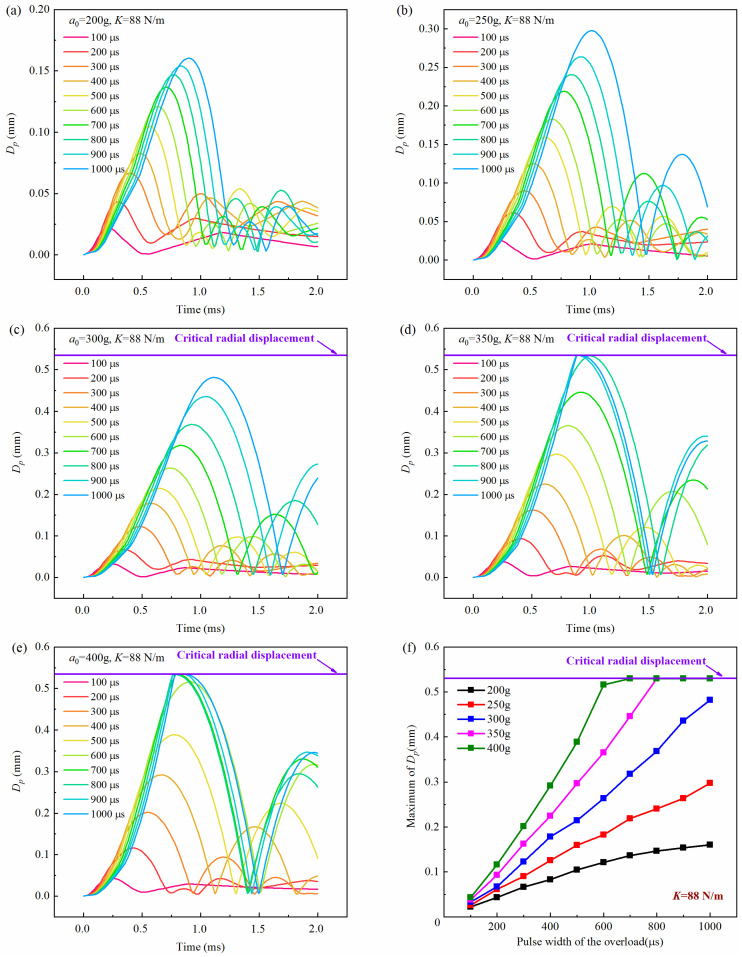
History curves of the free-end displacement of the impact rod of the inertial switch (*K* = 88 N/m) with time under different overload peak values with overload pulse width varying from 100 μs to 1000 μs: (**a**) *a*_0_ = 200 g, (**b**) *a*_0_ = 250 g, (**c**) *a*_0_ = 300 g, (**d**) *a*_0_ = 350 g, (**e**) *a*_0_ = 400 g and (**f**) relationship between maximum value of *D_p_* and overload pulse width. *D_p_* was the free-end radial displacement of the impact rod.

Figure 7, Figure 8 and Figure 9 show the history curves of the free-end displacement of the impact rod of the inertial switch under different loading conditions with different spring stiffness coefficients: *K* = 48 N/m, *K* = 68 N/m, and *K* = 88 N/m, respectively. It can be seen from the figures that the dynamic performance of the inertial switch was closely related to both the peak value and pulse width of the overload. Specifically, it can be seen from Figure 7 that, for the inertial switch with the spring stiffness coefficient *K* = 48 N/m, when the peak value was equal to 200 g, the inertial switch did not close in the pulse width range of 100 μs–1000 μs; when the peak value was equal to 250 g, the inertial switch started to close when the pulse width was not less than 800 μs; when the peak value was greater than 250 g (*a*_0_ = 300 g, 350 g, 400 g), the inertial switch closed reliably in the pulse width range of 600 μs–1000 μs. Therefore, the critical overload of the inertial switch with a spring stiffness coefficient *K* = 48 N/m could be determined as 250 g. Similarly, it can be seen from Figure 8 that, for the inertial switch with a spring stiffness coefficient *K* = 68 N/m, when the peak value was less than 300 g (*a*_0_ = 200 g, 250 g), the inertial switch did not close in the pulse width range of 100 μs–1000 μs; when the peak value was equal to 300 g, the inertial switch started to close when the pulse width was not less than 600 μs; when the peak value was greater than 300 g (*a*_0_ = 350 g, = 400 g), the inertial switch closed reliably in the pulse width range of 600 μs–1000 μs. Therefore, the critical overload of the inertial switch with a spring stiffness coefficient *K* = 68 N/m could be determined as 300 g. It can be seen from Figure 9 that, for the inertial switch with a spring stiffness coefficient *K* = 88 N/m, when the peak value was less than 350 g (a = 200 g, 250 g, 300 g), the inertial switch did not close in the pulse width range of 100 μs–1000 μs; when the peak value was equal to 350 g, the inertial switch started to close when the pulse width was in the range of 800 μs–1000 μs; when the peak value was greater than 350 g (*a*_0_ = 400 g), the inertial switch closed reliably in the pulse width range of 700 μs–1000 μs. Therefore, the critical overload of the inertial switch with a spring stiffness coefficient *K* = 88 N/m could be determined as 350 g. On the other hand, the maximum value of the free-end displacement of the impact rod *d_m_* and the corresponding time *t_r_* curves in Figure 7, Figure 8 and Figure 9 are extracted, as shown in Figure 7f, Figure 8f, Figure 9f, respectively. It can be seen from the figures that at the same peak value, with the increased pulse width of the overload, the value of *d_m_* illustrated a linear increasing trend; at the same pulse width, with the increasing peak value, the value of *d_m_* showed a gradually increasing trend, indicating that the peak value and the pulse width have a significant impact on the dynamic response of the inertial switch.

**Figure 10:**
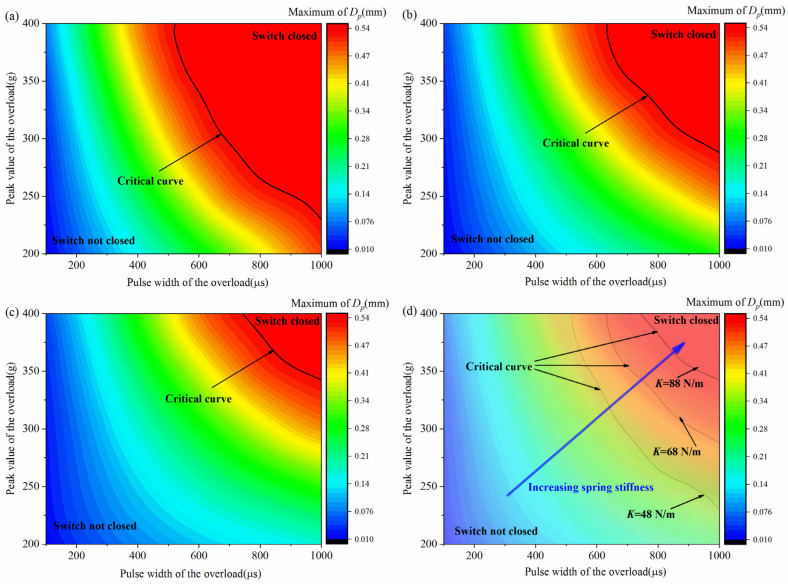
(**a**) Binary phase diagram of the overload pulse width and peak value of the inertial switch (*K* = 48 N/m), (**b**) binary phase diagram of the overload pulse width and peak value of the inertial switch (*K* = 68 N/m), (**c**) binary phase diagram of the overload pulse width and peak value of the inertial switch (*K* = 88 N/m), and (**d**) comparison of the critical overload peak value and pulse width curves of inertial switches with different spring stiffness coefficients. *D_p_* was the free-end radial displacement of the impact rod.

As can be seen from the results above, the peak value and the pulse width are two crucial factors for the dynamic performance of the inertial switch. Therefore, determining the critical peak value and pulse width is of great significance in the design process of the inertial switch. Based on the systematic simulation calculations, the maximum value of the free-end displacement of the impact rod under different peak values and pulse widths was extracted, and the binary phase diagrams of the peak value and the pulse width were then obtained, as shown in Figure 10. Accordingly, the corresponding critical peak value and pulse width could be determined, as shown by the black lines, laying a foundation for the design of the inertial switch.

## 5. Conclusions

In this work, a theoretical model of the inertial switch was proposed to calculate the free-end displacement. It was found that the theoretical prediction and numerical results are in good agreement. Furthermore, the critical overload peak value has great impact on the overload pulse width. The binary phase diagrams of the peak value and the pulse width were obtained based on a series of simulations, and the corresponding critical peak value and pulse width could then be determined.

In practical applications, the specific overload peak value and overload pulse width that the inertial switch experiences in the working environment should be determined first. Then, in combination with the binary phase diagram, the inertial switch with the corresponding spring coefficient could be easily selected. Furthermore, the inertial switch studied in this paper is widely applied in the military field, thus the vibration isolation performance will be the key focus of future research.

## Figures and Tables

**Table 1:** Comparisons between theoretical prediction and numerical results of the free-end displacement of the impact rod under *t*_0_ = 500 μs.

	*a*_0_ = 200 g		*a*_0_ = 300 g		*a*_0_ = 400 g	
	*d_m_*/mm	*t_r_*/μs	*d_m_*/mm	*t_r_*/μs	*d_m_*/mm	*t_r_*/μs
Theoretical prediction	0.18	670	0.35	840	0.53	780
Numerical simulation	0.17	680	0.36	860	0.53	740
Error	5.56%	1.49%	2.86%	2.38%	/	5.13%

Note: *d_m_* was the maximum value of the free-end displacement of the impact rod and *t_r_* was the corresponding response time.

**Table 2:** Comparisons between theoretical prediction and numerical results of the free-end displacement of the impact rod under *t*_0_ = 1000 μs.

	*a*_0_ = 200 g		*a*_0_ = 300 g		*a*_0_ = 400 g	
	*d_m_*/mm	*t_r_*/μs	*d_m_*/mm	*t_r_*/μs	*d_m_*/mm	*t_r_*/μs
Theoretical prediction	0.33	1000	0.53	910	0.53	720
Numerical simulation	0.36	1000	0.53	840	0.53	705
Error	9.09%	/	/	7.69%	/	2.08%

Note: *d_m_* was the maximum value of the free-end displacement of the impact rod and *t_r_* was the corresponding response time.

## Data Availability

The original contributions presented in the study are included in the article, further inquiries can be directed to the corresponding author.
